# *ERBB* family fusions are recurrent and actionable oncogenic targets across cancer types

**DOI:** 10.3389/fonc.2023.1115405

**Published:** 2023-04-24

**Authors:** Laura Schubert, Andrew Elliott, Anh T. Le, Adriana Estrada-Bernal, Robert C. Doebele, Emil Lou, Hossein Borghaei, Michael J. Demeure, Razelle Kurzrock, Joshua E. Reuss, Sai-Hong Ignatius Ou, David R. Braxton, Christian A. Thomas, Sourat Darabi, Wolfgang Michael Korn, Wafik S. El-Deiry, Stephen V. Liu

**Affiliations:** ^1^ Department of Medicine, Division of Medical Oncology, University of Colorado School of Medicine, Denver, CO, United States; ^2^ CARIS Life Sciences, Irving, TX, United States; ^3^ Department of Medicine, Division of Hematology, Oncology and Transplantation, University of Minnesota School of Medicine, Minneapolis, MN, United States; ^4^ Department of Hematology/Oncology, Fox Chase Cancer Center, Philadelphia, PA, United States; ^5^ Hoag Memorial Hospital Presbyterian, Center for Applied Genomic Technologies, Newport Beach, CA, United States; ^6^ Department of Medicine, University of California San Diego, La Jolla, CA, United States; ^7^ Department of Medicine, Georgetown University, Washington, DC, United States; ^8^ Department of Medicine, Division of Hematology/Oncology, University of California Irvine School of Medicine, Orange, CA, United States; ^9^ Hoag Memorial Hospital Presbyterian, Department of Pathology and Laboratory Medicine, Newport Beach, CA, United States; ^10^ New New England Cancer Specialists, Scarborough, ME, United States; ^11^ Department of Pathology and Laboratory Medicine, Cancer Center at Brown University, Providence, RI, United States; ^12^ Cancer Center at Brown University, Department of Pathology and Laboratory Medicine, Providence, RI, United States

**Keywords:** gene fusion, oncogene, EGFR, ERBB2, ERBB4 words: 3, 500 ERBB family fusions across cancer types

## Abstract

**Purpose:**

Gene fusions involving receptor tyrosine kinases (RTKs) define an important class of genomic alterations with many successful targeted therapies now approved for *ALK*, *ROS1*, *RET* and *NTRK* gene fusions. Fusions involving the *ERBB* family of RTKs have been sporadically reported, but their frequency has not yet been comprehensively analyzed and functional characterization is lacking on many types of *ERBB* fusions.

**Materials and methods:**

We analyzed tumor samples submitted to Caris Life Sciences (n=64,354), as well as the TCGA (n=10,967), MSK IMPACT (n=10,945) and AACR GENIE (n=96,324) databases for evidence of *EGFR, ERBB2* and *ERBB4* gene fusions. We also expressed several novel fusions in cancer cell lines and analyzed their response to EGFR and HER2 tyrosine kinase inhibitors (TKIs).

**Results:**

In total, we identified 1,251 *ERBB* family fusions, representing an incidence of approximately 0.7% across all cancer types. *EGFR, ERBB2*, and *ERBB4* fusions were most frequently found in glioblastoma, breast cancer and ovarian cancer, respectively. We modeled two novel types of *EGFR* and *ERBB2* fusions, one with a tethered kinase domain and the other with a tethered adapter protein. Specifically, we expressed *EGFR-ERBB4, EGFR-SHC1, ERBB2-GRB7* and *ERBB2-SHC1*, in cancer cell lines and demonstrated that they are oncogenic, regulate downstream signaling and are sensitive to small molecule inhibition with EGFR and HER2 TKIs.

**Conclusions:**

We found that *ERBB* fusions are recurrent mutations that occur across multiple cancer types. We also establish that adapter-tethered and kinase-tethered fusions are oncogenic and can be inhibited with EGFR or HER2 inhibitors. We further propose a nomenclature system to categorize these fusions into several functional classes.

## Introduction

Knowledge of the genetic landscape of cancers has grown increasingly critical to guide proper treatment choice. Chromosomal rearrangements involving receptor tyrosine kinases (RTKs) result in the aberrant activation of proliferative and pro-survival pathways. This is often accomplished through the contribution of a dimerization domain which allows constitutive activation of RTKs in the absence of ligand binding and/or enhanced expression from the new 5’ gene partner. Improved identification and understanding of these oncogenic fusions have resulted in the development of many successful targeted therapies over the last several years. *ALK, ROS1, NTRK, FGFR* and *RET* fusions now define an important subset of non-small cell lung cancers (NSCLC) and other malignancies with FDA-approved targeted inhibitors (1–9).

Activation of the ERBB/HER family of proteins has been repeatedly implicated in oncogenesis and can be induced by various molecular alterations, including *EGFR* point mutations or short insertions or deletions (indels) in NSCLC and *ERBB2* amplification in breast cancer (10, 11). HER family RTKs (EGFR, HER2, HER3 and HER4) are normally activated by ligand binding, dimerization and eventual activation of downstream pathways including RAS/MAPK and PI3K/AKT. HER family receptors can form both homodimers and heterodimers. In particular, HER2 and HER3 often form heterodimers because HER2 lacks a ligand binding domain and HER3 does not have a functional ATP binding pocket (12).

*ERBB* family fusions involving the *EGFR, ERBB2* and *ERBB4* genes are emerging therapeutic targets in several cancer types. Recurrent *EGFR-SEPT14* fusions have previously been identified in glioblastoma and are capable of activating proliferative signaling in the absence of EGFR ligands (13). Other *EGFR* fusions have also been rarely reported in lung adenocarcinoma as well as colorectal adenocarcinoma (14–16). Rearrangements of the *ERBB2* gene have been found in breast as well as gastric cancers and *ERBB4* fusions have been reported in lung adenocarcinomas and ovarian cancer cell lines (17–19). Fusions involving *ERBB* family ligands, such as *NRG1*, have also been identified in NSCLC as well as other cancer types (20, 21). Despite the identification of several gene fusions in this family, many of these have never been functionally validated as oncogenes *in vitro*.

In this study, we sought to comprehensively analyze the frequency and characteristics of *ERBB* fusions across cancer types. We queried a large cohort of patient samples profiled at Caris Life Sciences as well as several publicly available data sets (TCGA, MSK IMPACT and AACR GENIE) for *EGFR, ERBB2* and *ERBB4* fusions. We further investigated the biology of several novel *EGFR* and *ERBB2* fusions in an *in vitro* cell line model.

## Materials and methods

### Study cohort

Formalin-fixed paraffin-embedded (FFPE) patient samples (n=64,354) were submitted to a commercial CLIA-certified laboratory (Caris Life Sciences, Phoenix, AZ). The present study was conducted in accordance with guidelines of the Declaration of Helsinki, Belmont Report, and U.S. Common Rule. With compliance to policy 45 CFR 46.101(b) (4), this study was conducted using retrospective, de-identified clinical data. Therefore, the present study was considered IRB exempt, and patient consent was not required.

### Fusion detection by WTS

Gene fusion detection was performed on mRNA isolated from a FFPE tumor sample using the Illumina NovaSeq platform (Illumina, Inc., San Diego, CA) and Agilent SureSelect Human All Exon V7 bait panel (Agilent Technologies, Santa Clara, CA). FFPE specimens underwent pathology review to diagnose percent tumor content and tumor size; a minimum of 10% of tumor content in the area for microdissection was required to enable enrichment and extraction of tumor-specific RNA. Qiagen RNA FFPE tissue extraction kit was used for extraction, and the RNA quality and quantity was determined using the Agilent TapeStation. Biotinylated RNA baits were hybridized to the synthesized and purified cDNA targets and the bait-target complexes were amplified in a post capture PCR reaction. The resultant libraries were quantified, normalized and the pooled libraries are denatured, diluted and sequenced; the reference genome used was GRCh37/hg19 and analytical validation of this test demonstrated ≥97% Positive Percent Agreement (PPA), ≥99% Negative Percent Agreement (NPA) and ≥99% Overall Percent Agreement (OPA) with a validated comparator method.

### Fusion topology

Fusion topologies were assessed by mapping gene fusion sequences to the reference genome GRCh37/hg19 to identify chromosome number, sense/antisense strand, and genomic breakpoint coordinates for gene-pairs of each fusion event detected. For each tumor, detection of multiple isoforms of a unique gene-pair with the same 5’-3’ gene order, presumed to result from alternative splicing, and reciprocal fusion isoforms were scored as a single event in the topology distribution.

### Next-generation sequencing for 592-gene panel

NGS was performed on genomic DNA isolated from FFPE tumor samples using the NextSeq platform (Illumina, Inc., San Diego, CA). Matched normal tissue was not sequenced. A custom-designed SureSelect XT assay was used to enrich 592 whole-gene targets (Agilent Technologies, Santa Clara, CA). All variants were detected with >99% confidence based on allele frequency and amplicon coverage, with an average sequencing depth of coverage of >500X and an analytic sensitivity of 5%. Prior to molecular testing, tumor enrichment was achieved by harvesting targeted tissue using manual microdissection techniques. Genetic variants identified were interpreted by board-certified molecular geneticists and categorized as ‘pathogenic,’ ‘presumed pathogenic,’ ‘variant of unknown significance,’ ‘presumed benign,’ or ‘benign,’ according to the American College of Medical Genetics and Genomics (ACMG) standards. When assessing mutation frequencies of individual genes, ‘pathogenic,’ and ‘presumed pathogenic’ were counted as mutations while ‘benign’, ‘presumed benign’ variants and ‘variants of unknown significance’ were excluded.

The datasets presented in this article from Caris Life Sciences are not publicly available because the raw data is protected proprietary information – these datasets are available for qualified researchers upon reasonable request and with permission of Caris Life Sciences.

### Accession of public data sets

We queried the TCGA PanCancer Analyses, MSK Impact and AACR GENIE data bases through cBioPortal (accession dates 6/18/20, 8/8/20, 8/8/20 respectively) for *EGFR, ERBB2* and *ERBB4* fusions (22–25).

### Cell lines and reagents

H3122 and Ba/F3 cell lines were acquired through Dr. John Minna (The University of Texas Southwestern Medical Center, Dallas, TX) and Dr. Dan Theodorescu (University of Colorado Comprehensive Cancer Center, Aurora, CO), respectively. Cells were grown in RPMI-1640 with 10% FBS. The HEK293T cell line was purchased from ATCC. Alectinib was purchased from Selleck Chemicals. AKT pS437 (4058), AKT (2920), ERK pT202/Y204 (9101), ERK (9107), EGFR pY845 (6963) and HER2 pY877 (2241) were purchased from Cell Signaling Technology. EGFR (610017) and HER2 (610161) antibodies were purchased from BD Biosciences and anti-GAPDH was from Millipore.

### Lentiviral transduction

Lentiviral constructs for *EGFR* and *ERBB2* fusions were manufactured by Vector Builders Inc (Chicago, IL) in their pLV lentiviral vector under CMV promoters. Transduction into H3122 was performed as previously described (26) with the following additions: after puromycin selection, H3122 cells were cultured in the presence of 100nM alectinib to inhibit ALK signaling. Cell viability assays and signaling analysis *via* immunoblotting were performed in the presence of 100nM alectinib.

### Cell proliferation assays

Cell proliferation assays were performed using the CellTiter 96 Aqueous One Solution from Promega according to the manufacturer’s instructions after 72 hours of incubation with indicated inhibitors.

### Immunoblotting

Cells were lysed in T-PER (Thermo Scientific) with Halt Protease and Phosphatase Inhibitor (Thermo Scientific). Proteins were separated with SDS-PAGE, transferred to nitrocellulose membranes and stained with indicated antibodies. Secondary IR-Dye anti-mouse or anti rabbit IgG (LI-COR) antibodies were then added. Imaging was performed with the Odyssey Imager and Odyssey Image Studio software (LI-COR).

## Results

### Incidence

**Figure 1:**
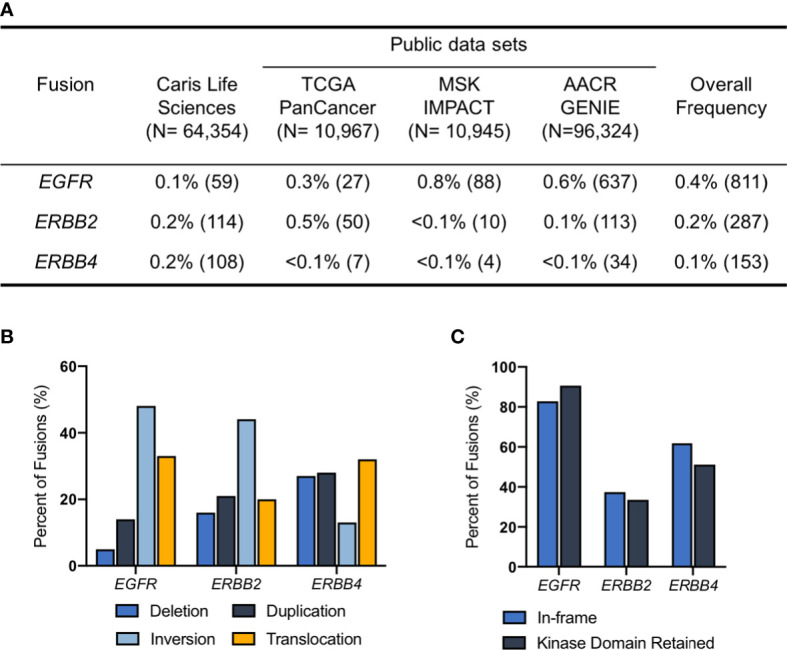
*ERBB* fusions are rare, recurrent alterations. **(A)** Frequency of *EGFR, ERBB2* and *ERBB4* fusions in Caris Life Sciences, TCGA PanCancer Atlas, MSK IMPACT and AACR GENIE datasets. **(B)** Fusion architecture in Caris Life Sciences datasets. **(C)** Percentage of *ERBB* fusions that are in-frame and/or have retained kinase domains in Caris Life Sciences datasets.

We identified a total of 1,251 *ERBB* family fusions across the four data sets including a total of 182,590 samples. *EGFR* fusions (n=811) were the most common overall, followed by *ERBB2* (n=287) and *ERBB4* fusions (n=153). We estimated that the frequency of *EGFR* fusions across data sets was 0.4%, *ERBB2* was 0.2% and *ERBB4* was 0.1% (**Figure 1A**). We did not assess the frequency of *ERBB3* fusions in this study, as the HER3 protein is unable to form functional homodimers (12). For the Caris samples, profiled by whole transcriptome sequencing, we further analyzed the topology of *ERBB* fusion transcripts to determine if the *ERBB* fusions resulted from deletion, inversion, duplication or translocation events. The majority of *EGFR* and *ERBB2* fusions were the result of inversions, while *ERBB4* fusions were most often translocations (**Figure 1B**). We also found that ~80% of *EGFR* fusions were both in-frame and retained a kinase domain. Approximately 50% of *ERBB4* and 40% of *ERBB2* fusions had these two features (**Figure 1C**).

### Fusion partners

Next, we analyzed the identity of the *ERBB* gene fusion partners. *EGFR-SEPT14* was the most common *EGFR* fusion, along with the reciprocal fusion *SEPT14-EGFR* that also results from the associated inversion event. However, based on the common breakpoint at *EGFR* exons 24-25, which retains the extracellular and kinase domains in the *EGFR-SEPT14* fusion, the *SEPT14-EGFR* reciprocal fusion would harbor only a small C-terminal portion of the EGFR protein, and thus, potentially have different or no oncogenic consequences compared to *EGFR-SEPT14* fusions. *ERBB2-IKZF3* and *ERBB2-PPP1R1B* were the two most common *ERBB2* fusions. *IKZF2-ERBB4* was the most common *ERBB4* fusion (**Table 1**).

**Table 1:** Recurrent *EGFR, ERBB2* and *ERBB4* fusions. Fusions observed more than once are reported above.

*EGFR* Fusions					
		Public data sets			
Fusion Partner	Caris LS	TCGA	MSK IMPACT	AACR GENIE	Total
*EGFR-SEPT14*	20	13	2	12	47
*SEPT14-EGFR*				17	17
*EGFR-VSTM2A*	3	1		2	6
*EGFR-PSPH*	4				4
*VOPP1-EGFR*	1			3	4
*SEC61G-EGFR*	2	2			4
*VSTM2A-EGFR*	1			2	3
*EGFR-LAMA2*				3	3
*EGFR-GBAS*				2	2
*EGFR-SEC61G*				2	2
*EGFR-TNS3*				2	2
*EGFR-VOPP1*				2	2
*ELDR-EGFR*				2	2
*ZNF713-EGFR*				2	2
*EGFR-GRB2*	2				2
*LANCL2-EGFR*	2				2
*ERBB2* Fusions					
		Public data sets			
Fusion Partner	Caris LS	TCGA	MSK IMPACT	AACR GENIE	Total
*ERBB2-IKZF3*	10	6		4	20
*ERBB2-PPP1R1B*	3	10		1	14
*ERBB2-CDK12*	10			2	12
*ERBB2-PGAP3*	10			2	12
*ERBB2-CTTN*		8			8
*GRB7-ERBB2*	2		2	4	8
*PGAP3-ERBB2*	8				8
*ERBB2-STARD3*	5			2	7
*GP2-ERBB2*	5				5
*C17orf37-ERBB2*			2	2	4
*ERBB2-PSMB3*		3			3
*JUP-ERBB2*	3				3
*ERBB2-GSDMA*	3				3
*ERBB2-TCAP*	1	1		1	3
*IKZF3-ERBB2*	1			2	3
*ERBB2-WIPF2*		1		1	2
*ERBB2-GRB7*	2				2
*ERBB2-GSDMB*	2				2
*RARA-ERBB2*			1	1	2
*ERBB2-SHC1*	2				2
*SHC1-ERBB2*			1	1	2
*ERBB2-IGFBP4*	2				2
*ERBB2-MED1*	2				2
*ERBB2-MED24*	2				2
*ERBB2-PLXDC1*	2				2
*ERBB2-RAPGEFL1*	2				2
*ERBB4* Fusions					
		Public data sets			
Fusion Partner	Caris LS	TCGA	MSK IMPACT	AACR GENIE	Total
*IKZF2-ERBB4*	20				20
*ERBB4-IKZF2*	13				13
*LANCL1-ERBB4*	3				3
*KANSL1L-ERBB4*	3				3
*ERBB4-TRIM33*				3	3
*ERBB4-PARD3B*			1	1	2
*ERBB4-PXMP2*			1	1	2
*AGAP1-ERBB4*	2				2
*KLF7-ERBB4*	2				2
*ERBB4-FN1*	2				2

### Cancer types

**Figure 2:**
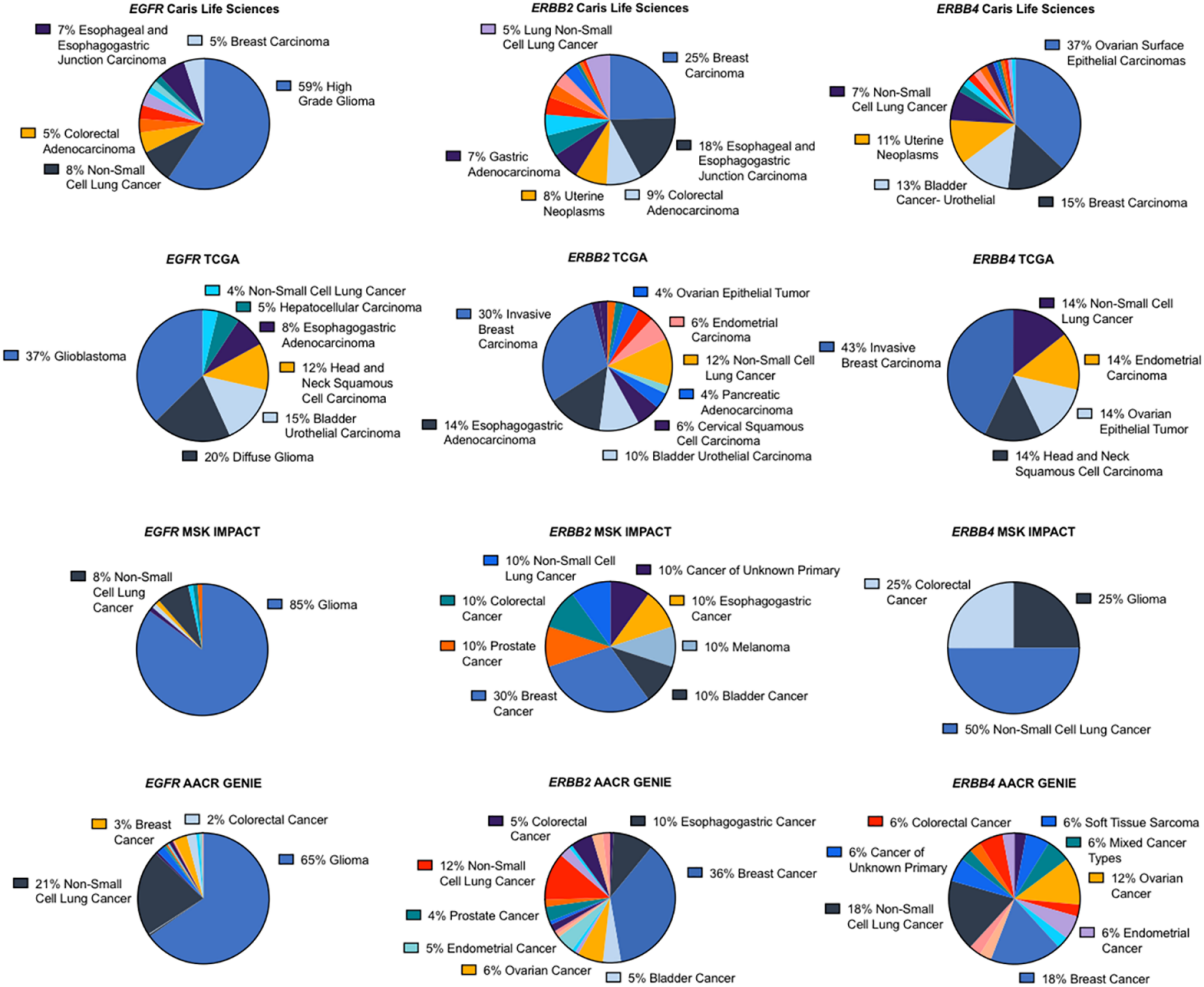
*ERBB* family fusions frequency by cancer type.

We assessed the most common cancer types harboring *ERBB* fusions. The majority of *EGFR* fusions were found in glioblastomas or gliomas, followed by NSCLC. *ERBB2* fusions were most frequently observed in breast cancer followed by gastroesophageal cancers. Depending on the dataset, *ERBB4* fusions were most often found in ovarian cancers, breast cancers or NSCLC (**Figure 2**).

### Fusion classes

**Figure 3:**
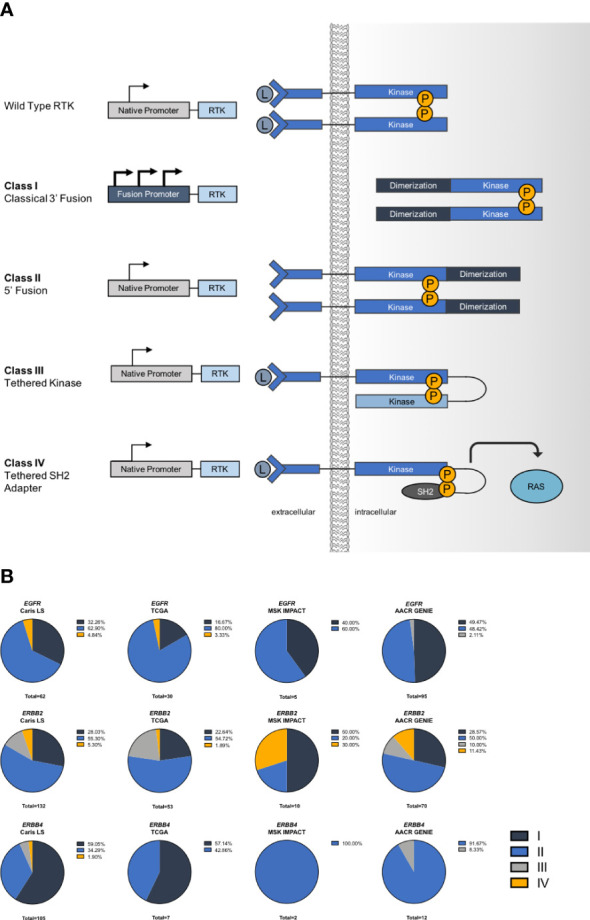
Mechanisms of activation in *ERBB* fusions. **(A)** Schematic describing classes of fusion oncogenes. **(B)** Frequency of each class of *ERBB* fusions.

We categorized the fusions that we identified based on their genomic architecture and proposed mechanism of activation. The first class, classical 3’ fusions, have a 5’ gene fusion partner that introduces a new 5’ promoter that likely enhances expression and typically has protein features, such as coiled-coil domains, that may promote dimerization of the fusion kinase. Class II fusions have a 3’ gene fusion partner that potentially allows constitutive dimerization. Class III fusions are tandemly linked to another kinase domain that may promote activation, presumably through enhanced dimerization. Lastly, Class IV fusions are tethered to an adapter protein that we propose allows direct activation of downstream pathways (**Figure 3A**). We assigned each fusion we identified to a class and found that overall Class II fusions were the most common among *ERBB* fusions, followed by Class I. Interestingly, we noticed that *ERBB2* fusions had an increased rate of Class III and class IV fusions, compared to *EGFR* or *ERBB4* fusions (**Figure 3B**).

### Co-occurring mutations

**Figure 4:**
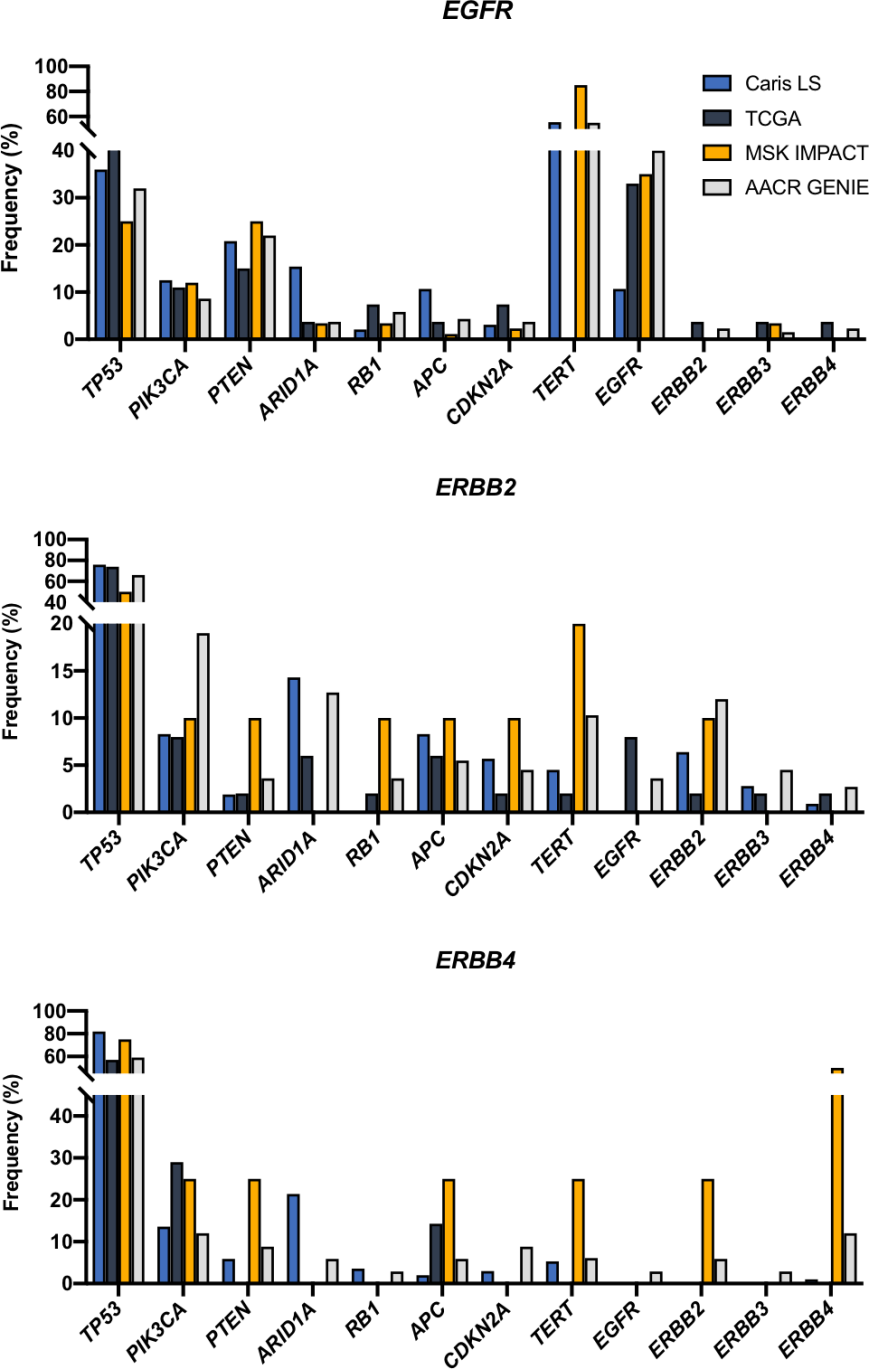
Co-occurring mutations in selected cancer-related genes.

We then analyzed which mutations co-occur with *ERBB* fusions. *EGFR* fusions frequently co-occurred with *TERT* mutations (>60%) and *EGFR* mutations (~30%). We also observed that *ERBB2* and *ERBB4* fusions co-occurred with *TP53* mutations (~60-80%) at a rate that was higher than the mutation rate found in the data sets queried (36-42%) (**Figure 4**).

### *ERBB* gene fusion cell line derivation

**Figure 5:**
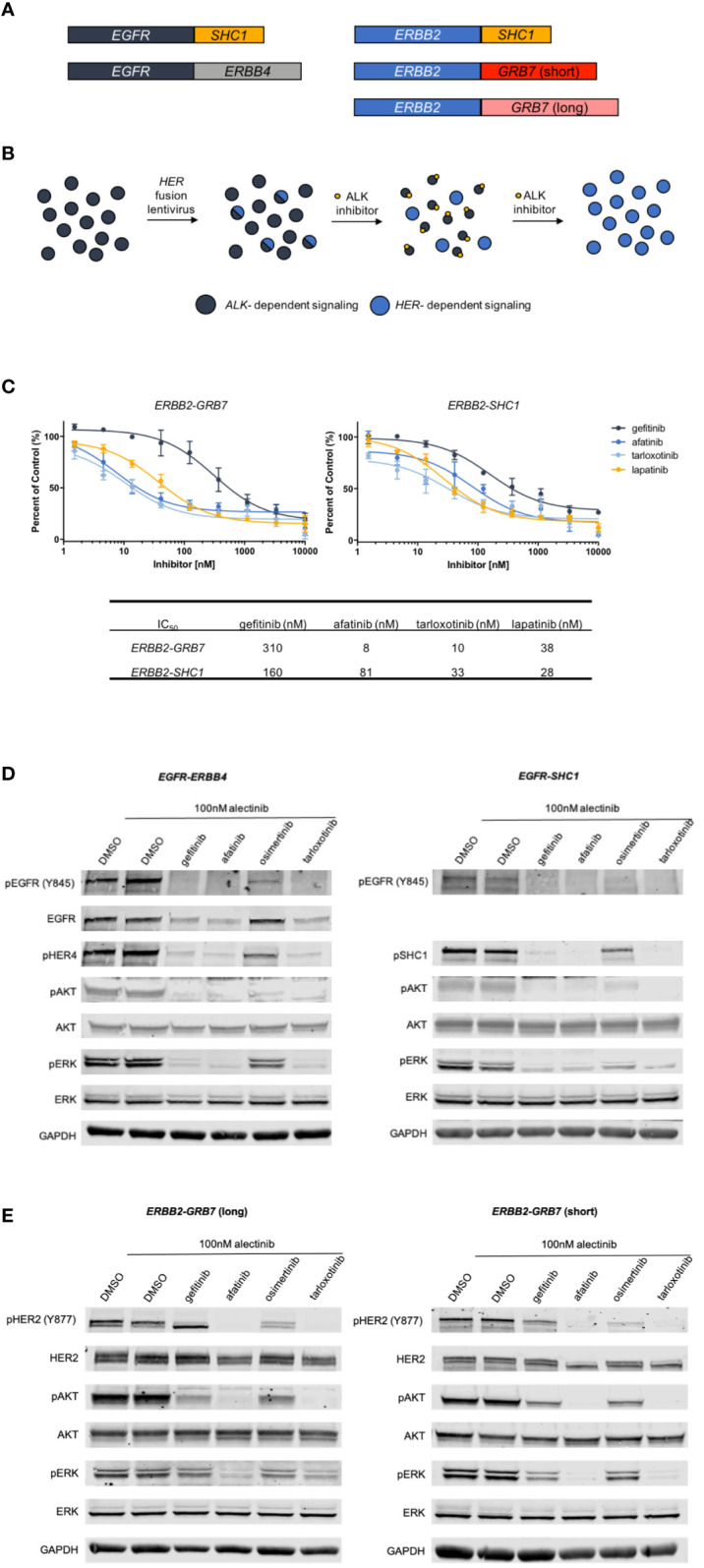
Derivation of *EGFR* and *ERBB2* fusion expressing cell lines. **(A)**. Schematic of *ERBB* fusions modeled in *in vitro* experiments. **(B)** Schematic describing the generation and selection of cells with *EGFR* or *ERBB2* fusions. *ALK*+ H3122 cells were transduced with *ERBB* fusion lentivirus, then treated with increasing doses of the ALK inhibitor alectinib to select for cells expressing *ERBB* fusions. **(C)** MTS proliferation assays of *ERBB2-GRB7* (short) and *ERBB2-SHC1* expressing cells treated with increasing concentrations of gefitinib, afatinib, tarloxotinib or lapatinib for two hours in the presence of 100nM alectinib to suppress ALK signaling. Error bars = ±SEM, N=3. **(D)** Western blot analysis of cells expressing *EGFR-ERBB4* and *EGFR-SHC1* fusions treated with 100nM alectinib and gefitinib, afatinib, osimertinib or tarloxotinib. **(E).** Western blot analysis of *ERBB2-GRB7* (short) or *ERBB2-SHC1* expressing cells treated with 100nM alectinib and 1000 nM gefitinib, 100nM afatinib, 100nM osimertinib or 100nM tarloxotinib for two hours.

Class I and Class II fusions have been extensively characterized and evidence supports oncogenic activity (13, 27, 28). Given the lack of functional characterization of Class III and IV fusions, we selected several of the *EGFR* and *ERBB2* fusions from the novel Class III and IV groups to analyze *in vitro*, including *EGFR-ERBB4* (Class III), *EGFR-SHC1* (Class IV), *ERBB2-SHC1* (Class IV) and *ERBB2-GRB7* (Class IV) (**Figure 5A**). First, we attempted to express these fusions in the Ba/F3 cell line, which is a murine B-cell cell line that is dependent on the addition of exogenous IL3 for proliferation but can become IL3-independent following oncogenic transformation. Ultimately, we were unable to transform the Ba/F3 cells with introduction of *ERBB* fusions. We hypothesized that because Ba/F3 cells may be lacking expression of signaling proteins necessary for these *ERBB* family fusions to successfully transform cells and therefore attempted to introduce *ERBB* fusions into a lung adenocarcinoma cell line. Previously we have demonstrated that H3122 can serve as model cells for novel fusions such as *NRG1* fusions (29). Parental H3122 cells without *ERBB* fusions are insensitive to EGFR or HER2 inhibition (29). Other groups have also used similar techniques to model novel oncogenes in lung adenocarcinoma cell lines (30). We transduced the novel *ERBB* fusions into the *EML4-ALK* fusion expressing H3122 cell line using lentivirus. We then performed short term selection with the ALK inhibitor alectinib to eliminate non-transduced cells and to select for cells containing *ERBB* fusions, which should inhibit proliferation of untransformed cells that remain dependent on ALK signaling, but not those cells expressing the *ERBB* fusions (**Figure 5B**). Once the cells were able to proliferate in 100nM alectinib we assessed their sensitivity to several EGFR/HER2 inhibitors. We found that both the *ERBB2-GRB7+* and *ERBB2-SHC1*+ cells were sensitive to the HER2 inhibitors afatinib, lapatinib and tarloxotinib suggesting both that the cells were now reliant on the *ERBB2* fusion for proliferation and that they are sensitive to HER2 inhibition. The transformed cells were less sensitive to the EGFR inhibitor gefitinib (**Figure 5C**).

### *ERBB* gene fusion cell signaling

Next, we characterized the signaling changes in our *ERBB+* cells in response to the EGFR inhibitors gefitinib, afatinib, osimertinib and tarloxotinib. Cells expressing either *EGFR-ERBB4* or *EGFR-SHC1* fusions showed a decrease in pEGFR following treatment, as well as decreased activation of the downstream signaling proteins pERK and pAKT. Additionally, we saw inhibition of the 3’ fusion partners pHER4 and pSHC1. As expected, the mutant-selective EGFR inhibitor osimertinib was less effective at inhibiting EGFR and downstream signaling, given the wild-type sequence of the *EGFR* kinase domain in these constructs (**Figure 5D**). We observed a similar pattern in our *ERBB2* fusion cell lines where treatment with HER2 inhibitors (afatinib and tarloxotinib) resulted in decreased HER2 phosphorylation and decreased pERK and pAKT. Gefitinib and osimertinib, which have less activity against HER2, resulted in less inhibition of HER2 and downstream signaling (**Figure 5E**).

## Discussion

We found that *ERBB* family fusions involving *EGFR, ERBB2* and *ERBB4* are rare, but that these recurrent alterations collectively represent a notable patient population that may benefit from available targeted therapies or those under development. We estimate that *ERBB* fusions may be present in up to 0.7% of cancers, which represents about 12,646 patients based on the 2020 cancer incidence of 1,806,569 cases (31). These fusions were detected across four different data sets and a wide range of cancer types. We believe that the synthesis of data from several different datasets allowed us to capture more rare genetic events and alterations that may be present in different types of sequencing analyses as well as build a cohort of samples that incorporates more rare diseases and patient population characteristics. Several TKIs now have demonstrated activity across cancer types, so called “agnostic” indications, including the TRK inhibitor larotrectinib, the TRK/ROS1 inhibitor entrectinib, and the RET inhibitors selpercatinib and pralsetinib (5, 6, 32–34). Consequently, larotrectinib and entrectinib have been granted tumor-agnostic FDA approvals and selpercatinib and pralsetinib are approved for both NSCLC and papillary or medullary thyroid cancers with RET fusions or mutations, respectively. FGFR inhibitors have similarly been approved for use in both cholangiocarcinoma and urothelial cancers with *FGFR* alterations (7–9). This highlights the potential utility of comprehensive NGS assays for patients that can broadly detect *ERBB* fusions, as many of these alterations are in theory targetable with currently available drugs or those in development.

When we analyzed the mutations that co-occurred with *ERBB* family fusions we observed that *ERBB2* and *ERBB4* fusions had a higher-than-average rate of *TP53* mutations while *EGFR* fusions had a lower-than-average rate of *TP53* mutations. This suggests that *TP53* mutations may be cooperative with *ERBB2* and *ERBB4* fusions and facilitate oncogenesis or provide a selective advantage. We also noticed many co-occurring *TERT* mutations in *EGFR* fusion samples, however, this is likely due to the high representation of glioblastomas, which frequently have *TERT* mutations (35). We also found that *EGFR* fusions co-occurred with *EGFR* point mutations in ~35% of samples.

We classified *ERBB* family fusions into several distinct classes based on genetic architecture and/or their proposed mechanism of activation. Most of the well characterized fusions, such as *ALK, NTRK, ROS1* and *RET* fall into Class I. Here, we describe several additional structural types of gene fusions. *EGFR* fusions were most commonly detected in glioblastoma or glioma, most of which were *EGFR-SEPT14* fusions. The majority of *EGFR* fusions we identified, including *EGFR-SEPT14*, were Class II (3’ fusions) which are likely activated by the addition of a dimerization domain. *EGFR-SEPT14* fusions have previously been reported in glioblastoma, where they can activate STAT3 signaling and are sensitive to treatment with lapatinib or erlotinib (13). This study suggested that Class II *EGFR* fusions are likely functional, activating oncogenic events.

We identified a unique class of fusions that are activated through tethering to either a kinase domain or an adapter protein (Class III and Class IV). Recruitment of adapter proteins is a crucial step in activation of downstream signaling *via* RTKs. We found that Class III and IV fusions were more highly represented in *ERBB2* fusions. We chose three adapter-linked fusions to model *in vitro: EGFR-SHC1, ERBB2-SHC1*, and *ERBB2-GRB7*. Both SHC1 and GRB7 are SH2 domain-containing adapter proteins responsible for activating MAPK signaling (36, 37). While these fusions have been previously reported, there have not been any functional studies of adapter protein-linked fusions (38). It is interesting to note that these fusions did not successfully transform Ba/F3 cells, suggesting that some *ERBB* fusions may require specific downstream proteins or expression profiles to transform cells. Because of this, we developed a novel approach for modeling oncogenic changes by using a cell line with a known driver oncogene (*ALK*) that is sensitive to an inhibitor (alectinib), which allowed us to select for cells containing *EGFR* or *ERBB2* fusions. We have previously employed a similar approach with CRISPR/Cas9 generated fusions in *ALK+* cells (29). We believe this approach will be widely useful and can be easily adapted for other cancer types or cell types and should be considered when model cell lines, such as Ba/F3, fail to support oncogenic transformation of putative novel oncogenes. These data suggest that there may only be certain cell types or conditions that are permissive for *ERBB* fusions to transform cells. Our data show that adapter-tethered *EGFR* and *ERBB2* fusions are capable of sustaining cancer cell growth and proliferation and, importantly, are sensitive to inhibition with EGFR and/or HER2 inhibitors. We also developed an *in vitro* model of a Class IV fusion, *EGFR-ERBB4*. We found that this fusion behaved similarly to the Class III fusions and was capable of regulating downstream signaling through pERK and pAKT. It is also interesting to note that while the *EGFR-ERBB4* chromosomal rearrangement contains *EGFR* exons 1-27, exons 26 and 27 are spliced out in the RNA transcript in order to maintain a proper reading frame. We have previously observed a similar mechanism in *ROS1* fusions (39) (**Supplementary Figure 2**).

Based on our *in vitro* data, we expect *ERBB* fusions can be successfully targeted with TKIs. These fusions do present a potential therapeutic challenge because they retain a wild-type, rather than mutated, kinase domain that may be more challenging to target specifically as has been the case with *EGFR* and *ERBB2* exon 20 mutations, which lack the significant therapeutic window observed for classical *EGFR* del 19 and L858R mutations. However, it has been previously shown that a patient with an *EGFR* kinase domain duplication, similar to the Class IV fusions, had a partial response to the EGFR inhibitor afatinib (40). Furthermore, there are two examples of patients with tumors harboring *EGFR-RAD51* fusions being responsive to afatinib or erlotinib (15, 16). The hypoxia-activated EGFR/HER2 inhibitor tarloxotinib, may be another mechanism of more specifically targeting *EGFR* or *ERBB2* fusions in only tumor cells (26). There is additional evidence that in patients harboring fusions, targeting other mutations without the fusion results in responses equivalent to patients with un-matched treatments; further suggesting that fusions are frequently key driver mutations (41). There is further evidence that gene fusions may also play an important role in tumor evolution and adaptation over time. Chromosomal instability is a hallmark of cancer that facilitates generation of new gene fusions, which are important genetic changes that enhance cellular fitness and facilitate oncogenesis (42). Furthermore, gene fusions may be a way to rapidly adapt to changes in the microenvironment; an area which warrants further study (43). Similarly gene fusions are already observed as resistance mechanisms to TKIs, further supporting their role in tumor evolution and response to changes in the microenvironment (29, 44).

Overall, we demonstrate that *EGFR, ERBB2* and *ERBB4* fusions are present across numerous cancer types and importantly are excellent candidates for targeted therapy development. We also propose a nomenclature system to help categorize fusion oncogenes. Finally, we show that the novel adapter-tethered and kinase-tethered fusions are oncogenic and sensitive to TKIs. These data show that *ERBB* family fusions are important therapeutic targets and warrant further study of their biology.

## Data availability statement

The datasets presented in this article from Caris Life Sciences are not publicly available because the raw data is protected proprietary information – these datasets are available for qualified researchers upon reasonable request and with permission of Caris Life Sciences.

## Author contributions

LS, AE, RD and SV contributed to the conception and design of the study. LS and AE performed data mining and analysis. AL and AE-B performed experiments. EL, HB, MD, RK, JR, SIO, DB, CT, SD, WK and WE-D performed data acquisition, analysis and interpretation. The first draft manuscript was written by LS. All authors contributed to the article and approved the submitted version.

## Supplementary material

Supplementary Figure 1
**(A)** MTS proliferation assay for cells expressing a longer variant of the *ERBB2-GRB7* fusion containing all exons for the *GRB7* gene. Cells were treated with increasing concentrations of gefitinib, afatinib, tarloxotinib or lapatinib for two hours. Error bars = ±SEM, N=3. **(B)** Western blot analysis of *ERBB2-GRB7* (long) expressing cells treated with 100nM alectinib and 1000 nM gefitinib, 100nM afatinib, 100nM osimertinib or 100nM tarloxotinib for two hours

Supplementary Figure 2
*EGFR* exons 26 and 27 are skipped to maintain reading frame in fusion transcript of *EGFR-ERBB4* fusion. Upper: Genomic sequencing of *EGFR-ERBB4* fusion including exons 1-27 of *EGFR* and exons 18-28 of *ERBB4*. Lower: RNA sequencing of *EGFR-ERBB4* fusion transcript and schematic illustrating skipping of exons 26 and 27.
