# Supplementary material for: ERBB family fusions are recurrent and actionable oncogenic targets across cancer types
Source: Front Oncol. 2023 Apr 24;13:1115405. doi: 10.3389/fonc.2023.1115405 (PMC10164992; doi:10.3389/fonc.2023.1115405)
Supplement: Supplementary Figure 1 — (A) MTS proliferation assay for cells expressing a longer variant of the ERBB2-GRB7 fusion containing all exons for the GRB7 gene. Cells were treated with increasing concentrations of gefitinib, afatinib, tarloxotinib or lapatinib for two hours. Error bars = ±SEM, N=3. (B) Western blot analysis of ERBB2-GRB7 (long) expressing cells treated with 100nM alectinib and 1000 nM gefitinib, 100nM afatinib, 100nM osimertinib or 100nM tarloxotinib for two hours [file Presentation_1.pptx]

## Slide 1
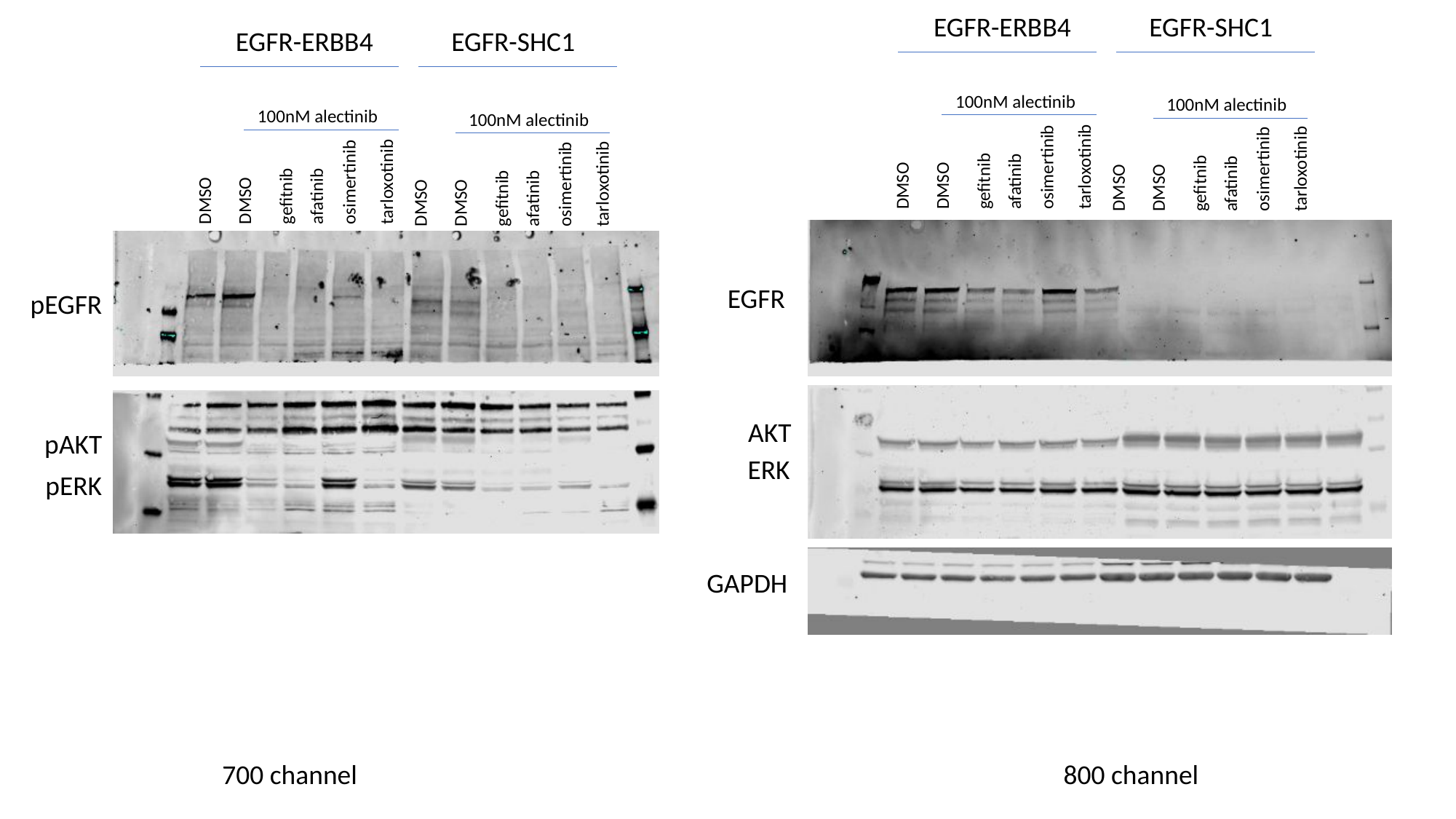

EGFR-ERBB4
EGFR-SHC1
EGFR-ERBB4
EGFR-SHC1
100nM alectinib
100nM alectinib
100nM alectinib
100nM alectinib
osimertinib
tarloxotinib
osimertinib
tarloxotinib
osimertinib
tarloxotinib
gefitnib
afatinib
osimertinib
tarloxotinib
gefitnib
DMSO
DMSO
afatinib
DMSO
DMSO
gefitnib
afatinib
gefitnib
DMSO
DMSO
afatinib
DMSO
DMSO
EGFR
pEGFR
AKT
pAKT
ERK
pERK
GAPDH
700 channel
800 channel

## Slide 2
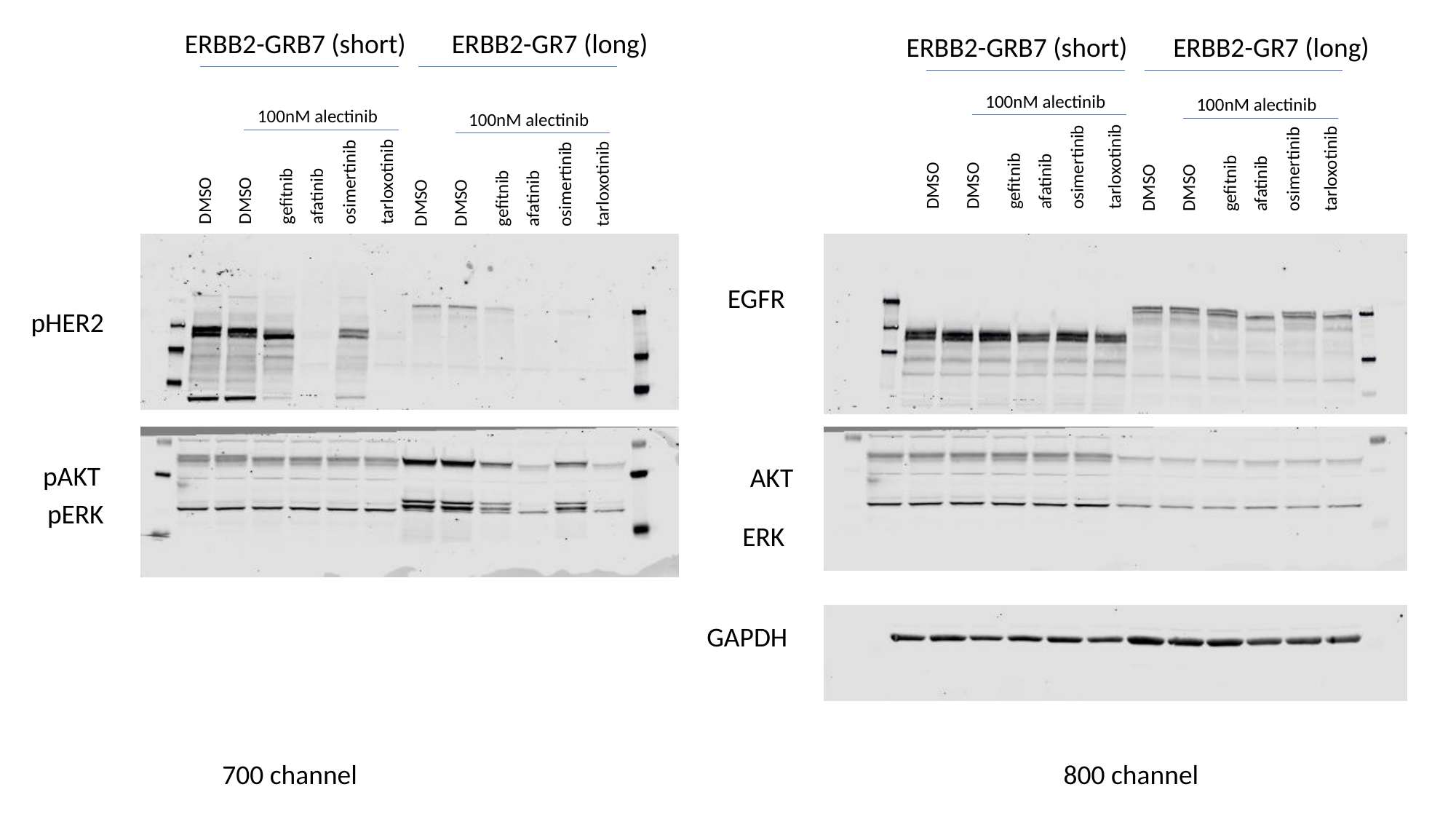

ERBB2-GRB7 (short)
ERBB2-GR7 (long)
ERBB2-GRB7 (short)
ERBB2-GR7 (long)
100nM alectinib
100nM alectinib
100nM alectinib
100nM alectinib
osimertinib
tarloxotinib
osimertinib
tarloxotinib
osimertinib
tarloxotinib
gefitnib
afatinib
osimertinib
tarloxotinib
gefitnib
DMSO
DMSO
afatinib
DMSO
DMSO
gefitnib
afatinib
gefitnib
DMSO
DMSO
afatinib
DMSO
DMSO
EGFR
pHER2
pAKT
AKT
pERK
ERK
GAPDH
700 channel
800 channel
